# Supplementary material for: Blood Meal Sources of Anopheles spp. in Malaria Endemic Areas of Honduras
Source: Insects. 2020 Jul 16;11(7):450. doi: 10.3390/insects11070450 (PMC7412045; doi:10.3390/insects11070450)
Supplement: Supplementary file 1 [file insects-11-00450-s001.pdf]

Supplementary Table 1 *Anopheles* specimen collection sites in Honduras and number of malaria cases during 2018.

Department	Municipality	Coordinates	Altitude (m.a.s.l.)	Month of collection	Malaria cases in 2018 by department
Atlántida	La Ceiba	15.748587, -86.900546	7	February	20
Atlántida	La Ceiba	15.758790, -86.867092	7	February	
Colón	Iriona	15.938416, -85.058888	4	March	100
Colón	Iriona	15.773889, -85.134556	27	March	
Colón	Sonaguera	15.629846, -86.287587	82	April	
Colón	Tocoa	15.655448, -86.04725	38	April	
El Paraíso	Morocelí	14.103168, -86.917882	600	August	33
Comayagua	Comayagua	14.439279, -87.689953	588	August	18
Gracias a Dios	Tikirraya	15.018379, -83.641264	13	October	260
Gracias a Dios	Kaukira	15.309131, -83.565868	8	October	
